# MicroRNA-21 Limits Uptake of *Listeria monocytogenes* by Macrophages to Reduce the Intracellular Niche and Control Infection

**DOI:** 10.3389/fcimb.2017.00201

**Published:** 2017-05-23

**Authors:** Daniel G. W. Johnston, Jay Kearney, Zbigniew Zasłona, Michelle A. Williams, Luke A. J. O'Neill, Sinéad C. Corr

**Affiliations:** ^1^School of Biochemistry and Immunology, Trinity Biomedical Sciences Institute, Trinity College DublinDublin, Ireland; ^2^Department of Microbiology, Moyne Institute of Preventive Medicine, School of Genetics and Microbiology, Trinity College DublinDublin, Ireland

**Keywords:** microRNA, *Listeria*, macrophage, phagocytosis, miR-21, MARCKS

## Abstract

MiRNAs are important post-transcriptional regulators of gene expression. MiRNA expression is a crucial part of host responses to bacterial infection, however there is limited knowledge of their impact on the outcome of infections. We investigated the influence of miR-21 on macrophage responses during infection with *Listeria monocytogenes*, which establishes an intracellular niche within macrophages. MiR-21 is induced following infection of bone marrow-derived macrophages (BMDMs) with *Listeria*. MiR-21^−/−^ macrophages display an increased bacterial burden with *Listeria* at 30 min and 2 h post-infection. This phenotype was reversed by the addition of synthetic miR-21 mimics to the system. To assess the immune response of wildtype (WT) and miR-21^−/−^ macrophages, BMDMs were treated with bacterial LPS or infected with *Listeria*. There was no difference in IL-10 and IL-6 between WT and miR-21^−/−^ BMDMs in response to LPS or *Listeria*. TNF-α was increased in miR-21^−/−^ BMDMs stimulated with LPS or *Listeria* compared to WT macrophages. We next assessed the production of nitric oxide (NO), a key bactericidal factor in *Listeria* infection. There was no significant difference in NO production between WT and miR-21^−/−^ cells, indicating that the increased bacterial burden may not be due to impaired killing. As the increased bacterial load was observed early following infection (30 min), we questioned whether this is due to differences in uptake of *Listeria* by WT and miR-21^−/−^ macrophages. We show that miR-21-deficiency enhances uptake of FITC-dextran and FITC-*Escherichia coli* bioparticles by macrophages. The previously observed *Listeria* burden phenotype was ablated by pre-treatment of cells with the actin polymerization inhibitor cytochalasin-D. From analysis of miR-21 targets, we selected the pro-phagocytic regulators myristoylated alanine-rich C-kinase substrate (MARCKS) and Ras homolog gene family, member B (RhoB) for further investigation. MARCKS and RhoB are increased in miR-21^−/−^ BMDMs, correlating with increased uptake of *Listeria*. Finally, intra-peritoneal infection of mice with *Listeria* led to increased bacterial burden in livers of miR-21^−/−^ mice compared to WT mice. These findings suggest a possible role for miR-21 in regulation of phagocytosis during infection, potentially by repression of MARCKS and RhoB, thus serving to limit the availability of the intracellular niche of pathogens like *L. monocytogenes*.

## Introduction

Macrophages are important effector cells of the innate immune system and represent the first line of defense against invading bacterial pathogens (Benoit et al., 2008). Professional phagocytic cells of the innate immune system, macrophages encounter and engulf invading pathogens, cellular debris and other potential deleterious substances. Their expression of pattern recognition receptors (PRRs) (Takeuchi and Akira, 2010), both on the cell membrane and in the cytosol, allows them recognize potentially harmful bacteria. Following recognition of invading bacteria, intracellular signaling pathways are initiated leading to actin polymerization and formation of the phagocytic cup which subsequently encloses around the bacterium to form the phagosome. The phagosome subsequently undergoes a series of maturation steps which involves fusion with endosomal vesicles and fission vesicles, moving through early, intermediate and late stages culminating in formation of the mature phagolysosome which has acquired the full bactericidal repertoire (Weiss and Schaible, 2015). These include ability to generate reactive nitrogen intermediates such as nitric oxide (NO) and production of reactive oxygen species (ROS). In this way, macrophages play a critical role in host responses to intracellular pathogens and the clearance of infections which significantly contribute to the high morbidity and mortality rates associated with infectious diseases worldwide. However, certain intracellular bacteria have evolved strategies which allow them to exploit these intracellular niches. *Listeria monocytogenes* is the causative agent of the group of systemic infections known as listeriosis, associated with a fatality rate of 20% or more and the third leading cause of death among food-borne bacteria (Ramaswamy et al., 2007). *Listeria*'s ability to establish itself intracellularly where it can avoid host responses, creates a more favorable environment that ensures their pathogenesis. Indeed, *L. monocytogenes* have evolved to escape from the phagolysosome through the expression of a hemolysin, LLO, and subsequently grow and replicate within the cytosol of macrophages. The ability of *Listeria* to establish an intracellular niche and evade immune surveillance typifies the struggle between infectious agents and the host immunity and is critical to the outcome of infection (Corr and O'Neill, 2009).

MiRNA have emerged as critical regulators of host immune responses. MiRNA are short, non-coding RNAs that have been shown to affect numerous cellular processes in a post-transcriptional manner (Bartel, 2004; He and Hannon, 2004; Almeida et al., 2011). The role of miRNAs in immunity has been an area of intense research in recent years, and many have been implicated in the regulation of immune cell function including the fine-tuning of PRR signaling (Baltimore et al., 2008; O'Neill et al., 2011; Quinn and O'Neill, 2011). Although, there is growing understanding that regulation of miRNA expression is a crucial part of the host response to bacterial infection, knowledge of their cellular expression in response to bacteria and the impact of this on the outcome of infections is limited. Furthermore, modulation of miRNAs has emerged as a novel strategy employed by bacterial pathogens to manipulate host cell pathways and survive within host cells. MiR-21 is one of the most highly expressed miRNAs in many mammalian cell types (Krichevsky and Gabriely, 2009). MiR-21 is induced by inflammatory stimuli in particular in myeloid cells including monocytes, macrophages and dendritic cells, however the functional outcome of this is not well characterized. However, gradually a picture has developed of miR-21 as an anti-inflammatory miRNA that serves to curb excessive responses and begin the resolution phase of inflammation. It was shown to regulate expression of the anti-inflammatory cytokine IL-10 in macrophages in response to bacterial LPS, by targeting PDCD4, a negative regulator of IL-10 translation (Sheedy et al., 2010; Sheedy, 2015). A study of asthma showed that miR-21 negatively regulates immune responses in dendritic cells, by controlling the production of pro-inflammatory IL-12 (Lu et al., 2009, 2011). In addition, miR-21 has also been implicated in positively regulating the phenomenon of efferocytosis whereby activated macrophages alter their behavior to take up dying cells and prevent further inflammation (Das et al., 2014). MiR-21 has previously been shown under certain contexts to act as a break in the differentiation of macrophages to an M2-like phenotype, allowing a more robust bactericidal M1 macrophage to emerge. Although the role of miR-21 in the host response to bacterial pathogens is relatively unexplored, this implies a potentially important role for miR-21 in the control of infection (Wang et al., 2015).

In the current study, we sought to elucidate the importance of miR-21 during infection, in particular to regulate the immune response to *L. monocytogenes*. The ability of pathogens to establish an intracellular niche is dependent in part, on their uptake by macrophages. Here we show that miR-21 is induced in response to infection of macrophages with *L*. monocytogenes to regulate the amount of phagocytosis thereby limiting the intracellular niche of this pathogen. To our knowledge, this is the first report of a role for miR-21 as a host-strategy to curb infection with *L. monocytogenes*.

## Materials and methods

### Bacterial strains and growth conditions

*Listeria monocytogenes* EGDe (BUG1600, ATCC BAA-679) were grown in brain heart infusion (BHI, Oxoid), aerobically at 37°C shaking (200 rpm). All experiments performed using *L. monocytogenes* were performed in ClassII Biohazard facilities.

### Mice

MiR-21-deficient (miR-21^−/−^) mice were developed by Taconic Artemis using a Cre/*lox* approach. Briefly, miR-21 was modified by the insertion of two loxP sites that enable excision of the floxed miR-21 segment through Cre-mediated recombination. Chimeric offspring were backcrossed onto the C57BL/6J background for a total of 8 generations. Homozygous deletion of miR-21 was confirmed by PCR genotyping. Homozygous miR-21^−/−^ and WT littermates were used for animal studies. Animals were maintained in ventilated cages at 21 ± 1°C, humidity 50 ± 10% and with a 12 h-light/12 h-dark light cycle under specific pathogen-free conditions, in line with Irish and European Union regulations. Food and water were available *ad libitum* throughout all of the experiments. All experiments involving use of mice or mouse tissue were subject to ethical approval by the Animal Research Ethics Committee (AREC), a Level 2 ethics committee responsible for reviewing the proposed use of animals in teaching and research at Trinity College Dublin, and were carried out in accordance with the recommendations of the Irish Health Products Regulatory Authority, the competent authority responsible for the implementation of Directive 2010/63/EU on the protection of animals used for scientific purposes in accordance with the requirements of the S.I No 543 of 2012.

### Isolation of bone marrow-derived macrophages

Tibia and femur from 6 to 8 week-old C57BL/6J and genetically-matched miR-21^−/−^ mice were collected in ice cold PBS. Bones were sterilized with 70% ethanol, cleaned and flushed with a 25-G needle using cold DMEM (Gibco) supplemented with 10% FCS and 1% penicillin-streptomycin (Sigma Aldrich). Following red-blood cell lysis, cells were seeded onto non-cell culture coated 10 cm dishes in complete DMEM containing 20% M-CSF containing L929 media and incubated 37°C with 5% CO_2_ for 6 days. Subsequently, BMDMs were seeded at 5 × 10^5^ cells/ml in 12-well tissue culture plates (Sarstedt) in DMEM containing 10% L929 and 10% FCS.

### Isolation of resident peritoneal macrophages

Resident macrophages, peritoneal exudate cells (PECs) were isolated from 6 to 8 week-old C57BL/6J and genetically-matched miR-21^−/−^ mice by washing the peritoneum with 3 mL sterile PBS. The recovered cells were centrifuged at 300 g for 5 min and resuspended in complete medium. 12-well tissue culture plates were seeded at 5 × 10^5^ cells/ml (Sarstedt) in DMEM containing 10% FCS.

### Transfection of bone marrow-derived macrophages

BMDMs were seeded at 3 × 10^5^ cells/ml in 12-well tissue culture plates (Sarstedt) in DMEM containing 10% L929 and 10% FCS. The following day cells were transfected with 20 nmoles Mission® miR-21 mimic or negative control RNA (Sigma) for 24 h using RNAiMax transfection reagent (ThermoFisherScientific).

### Phagocytosis/cellular uptake assays

Twenty-four hour following seeding, *L. monocytogenes* were added at MOI 100:1 for 15 min, and media subsequently replaced with DMEM containing gentamicin (100 mg/ml). At 0.5 or 2 h, monolayers washed and lysed and subsequently plated on to BHI agar plates for determination of rates of phagocytosis, expressed as Log CFU/ml (or as CFU/cell as in Supplementary Figure 1). For FITC-dextran uptake assays, BMDMs or PECs were plated at 5 × 10^5^ cells/ml, in a 12-well tissue culture plate (Sarstedt) for 24 h. Cells were then incubated with 1 mg/ml FITC-dextran at 37°C for 1 h. Control cells were left untreated, or were treated and incubated at 4°C. At the end of the incubation, the cells were collected for assessment of FITC-dextran uptake by flow cytometry (BD Fortessa flow cytometer). For Vybrant™ Phagocytosis assays (Molecular Probes), BMDMs were plated at 5 × 10^5^ cells/ml in a 96-well plate and incubated with FITC-*E. coli* Bioparticles® for 2 h according to the manufacturer's instructions before extracellular FITC was quenched using trypan blue and fluorescence assayed using a FLUOstar Optima microplate reader at emission ~520 nm and excitation ~480 nm. Cytochalasin-D was used at 10 μM for 30 min pre-treatment in some assays.

### Enzyme linked immunosorbent assay (ELISA)

BMDMs were treated with LPS (Alexis) at 100 ng/ml or infected with *L. monocytogenes* at MOI 100:1 for 24 h. After an initial infection with *L. monocytogenes* for 15 min, media was subsequently replaced with DMEM containing gentamicin (100 mg/ml). Murine IL-10, IL-6, and TNF-α production was detected in macrophage supernatants by ELISA according to the manufacturer's procedure (R&D Duoset). Optical density was measured at 450 nm and cytokine concentrations were determined using a standard curve, expressed as pg/ml.

### Quantitative real-time PCR (qPCR)

Total RNA was extracted from BMDMs using a PureLink RNA extraction kit (Applied Biosystems) according to the manufacturer's instructions. Total RNA was reverse transcribed with a high-capacity cDNA archive kit (Applied Biosystems) and the cDNA was amplified using both Taqman and SYBR green-based real-time PCR on a 7,300 real-time PCR system (Applied Biosystems). Primer sequences are listed in Table 1. Relative quantification (RQ) of mRNA levels were determined by the 2^−ΔΔCT^ method comparing genes of interest to endogenous controls (U6 or Rps13). MiR-21 and U6 primers and probes were obtained from Applied Biosystems.

**Table 1:** Primers used for Sybr-Green qPCR^*^.

**Primer pair**	**Forward**	**Reverse**
MARCKS	5′-CTCCTCCTTGTCGGCGGCCGG-3′	5′-GGCCACGTAAAAGTGAACGGC-3′
RhoB	5′-GACGGCAAGCAGGTGGAG-3′	5′-ATGGGCACATTGGGGCAG-3′
Rps13	5′-GGCCCACAAGCTCTTTCCTT-3′	5′-GACCTTCTTTTTCCCGCAGC-3′

### Nitric oxide (NO) assay

To estimate NO release in response to *L. monocytogenes*, BMDMs were infected at MOI 100:1 for 15 min, the media was then replaced with DMEM containing gentamicin (100 mg/ml) for a further 1 h 45 min or 23 h and 45 min, and subsequently the nitrate present in the supernatants of macrophages at 2 h post-infection was measured using a Griess reaction according with the manufacturer's instructions (Promega). To estimate NO release in response to LPS, BMDMs were treated with LPS (100 ng/ml) for 2 or 24 h. Optical density was read between 520 and 550 nm and the nitrate present in each sample was quantified using a standard curve.

### Immunoblotting

BMDM cell lysates were obtained following infection with *L. monocytogenes* or from untreated samples. BMDMs were infected at MOI 100:1 for 15 min, and the media was then replaced with DMEM containing gentamicin (100 mg/ml) for a further 105 min. Samples were clarified, denatured with SDS loading buffer, and boiled for 5 min. A total of 40 mg protein lysate was fractionated on 12% SDS-PAGE, transferred to polyvinylidene fluoride membranes (Millipore) and probed with primary Abs to murine MARCKS (1:2,000 dilution, ab51100, Abcam), murine RhoB (1:1,000 dilution, sc-180, Santa Cruz) or murine β-actin (1:10,000 dilution, Clone AC-74, Sigma), incubated with horseradish peroxidase-conjugated secondary antibodies (Santa Cruz Biotechnology) and visualized using WesternBright ECL HRP substrate (Advansta), BD ChemiDoc system and ImageLab software.

### *In vivo L. monocytogenes* infection

Mice were intra-peritoneally or orally infected with *L. monocytogenes* at 1 × 10^6^ CFU or 5 × 10^7^ CFU respectivly. 3 days of 6 days p.i., respectively, livers were harvested and homogenized in PBS. Serial dilutions of this homogenate were plated on to BHI agar plates for determination of dissemination levels. Levels were expressed as Log CFU/liver.

### MiRNA database analysis

A combination of miRBase, miRWALK2.0 and TargetScan were used to identify potential miR-21 targets along with consultation of the literature.

### Statistical analysis

Numerical results are given as arithmetic means ± standard deviations. Statistical differences were analyzed by GraphPad Prism 5.0 statistical software (GraphPad Software Inc., San Diego, USA) or Student's *t*-test. *P*-values of less than 0.05 (*p* ≤ 0.05) are considered statistically significant.

## Results

### MiR-21 is induced during infection of macrophages with *Listeria* and its expression influences bacterial burden

**Figure 1:**
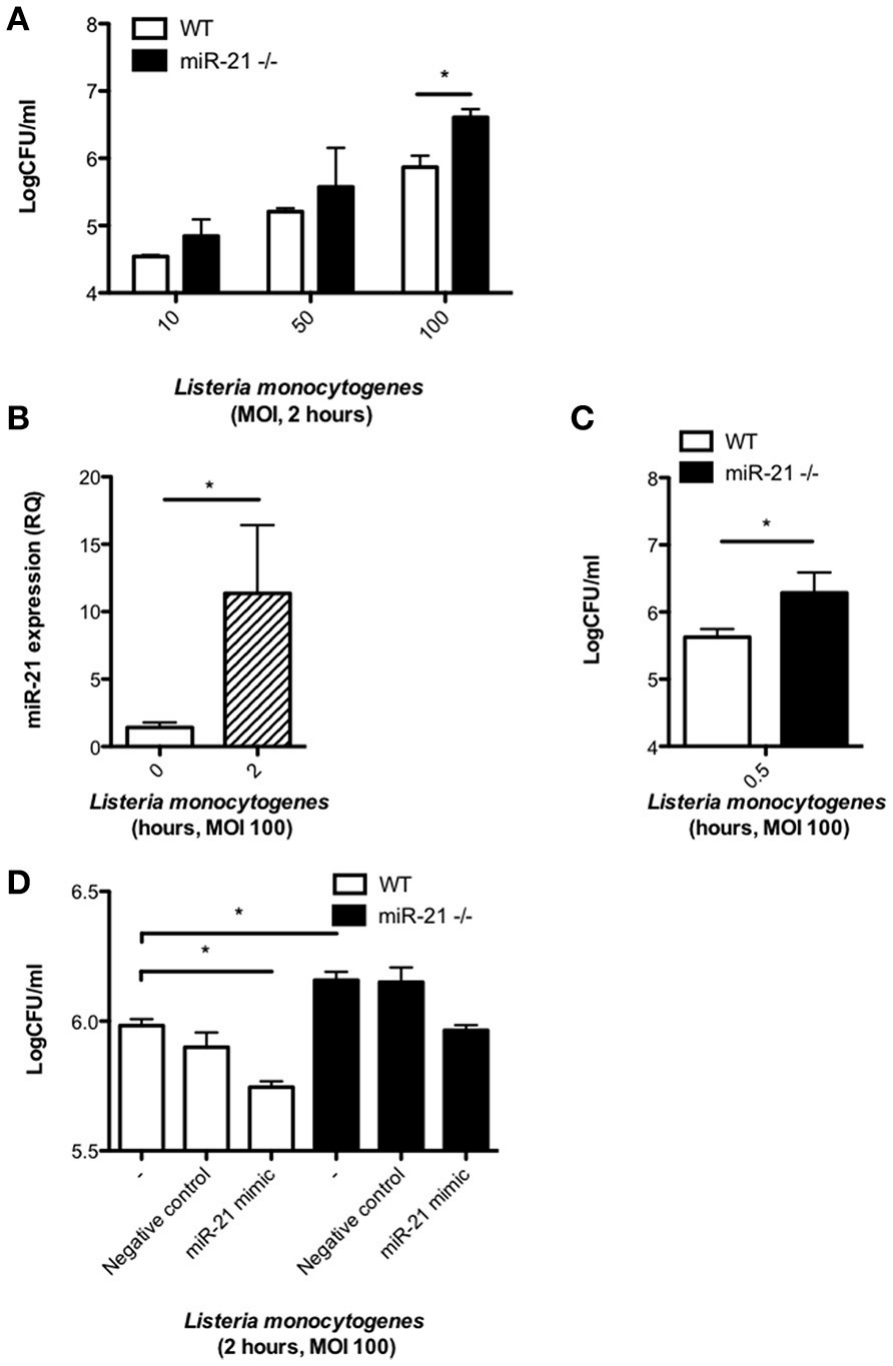
**MiR-21 is induced during *L. monocytogenes* infection of macrophages and its expression influences bacterial burden**. BMDMs were infected with *L. monocytogenes* at MOIs of 10, 50, and 100 for 15 min and subsequently cultured with media containing gentamicin (100 ug/ml). **(A)** At 2 h post-infection, the number of intracellular bacteria in WT and miR-21^−/−^ BMDMs was determined and expressed as Log CFU/ml. **(B)** At 2 h post-infection the RNA was isolated from WT BMDMs and mature miR-21 expression was assayed by qPCR and data expressed as relative expression from triplicate samples. **(C)** At 0.5 h the number of intracellular bacteria in WT and miR-21^−/−^ BMDMs was determined and expressed as Log CFU/ml. **(D)** WT and miR-21^−/−^ BMDMs were transfected with a miR-21 mimic or negative control for 24 h before subsequent *Listeria* infection. At 2 h post-infection, the number of intracellular bacteria was determined and expressed as Log CFU/ml. Data are expressed as means ± SD, *n* = 3 (except D where *n* = 2 for miR-21^−/−^ BMDMs), and are representative of at least three independent experiments. ^*^Designates a *p-*value < 0.05 by Students *t*-test.

Previous studies have shown that miR-21 expression in macrophages is induced by stimulation with bacterial TLR agonists such as LPS (Sheedy et al., 2010). To gain insight into the role of miR-21 during infection, we infected WT and miR-21^−/−^ BMDMs with the intracellular bacterial pathogen *L. monocytogenes* and assayed the bacterium's intracellular survival. Strikingly, BMDMs deficient for miR-21 had a significantly higher bacterial burden than WT macrophages after 2 h infection with high bacterial loads (Figure 1A). MiR-21 expression was significantly induced in WT BMDMs upon infection with *L. monocytogenes* (Figure 1B). The difference in bacterial burden observed after 2 h was also apparent after only 30 min infection (Figure 1C). In order to confirm miR-21's importance in *L. monocytogenes* infection, WT and miR-21^−/−^ BMDMs were transfected with synthetic miR-21 mimics, and a significant reduction of bacterial burden was observed in WT cells, as well as a reduction of bacterial burden in the miR-21^−/−^ cells toward WT levels (Figure 1D). These results suggest that miR-21 plays an important role during infection and aids control of *Listeria* by macrophages.

### Loss of MiR-21 alters cytokine expression in response to LPS but not *Listeria*

**Figure 2:**
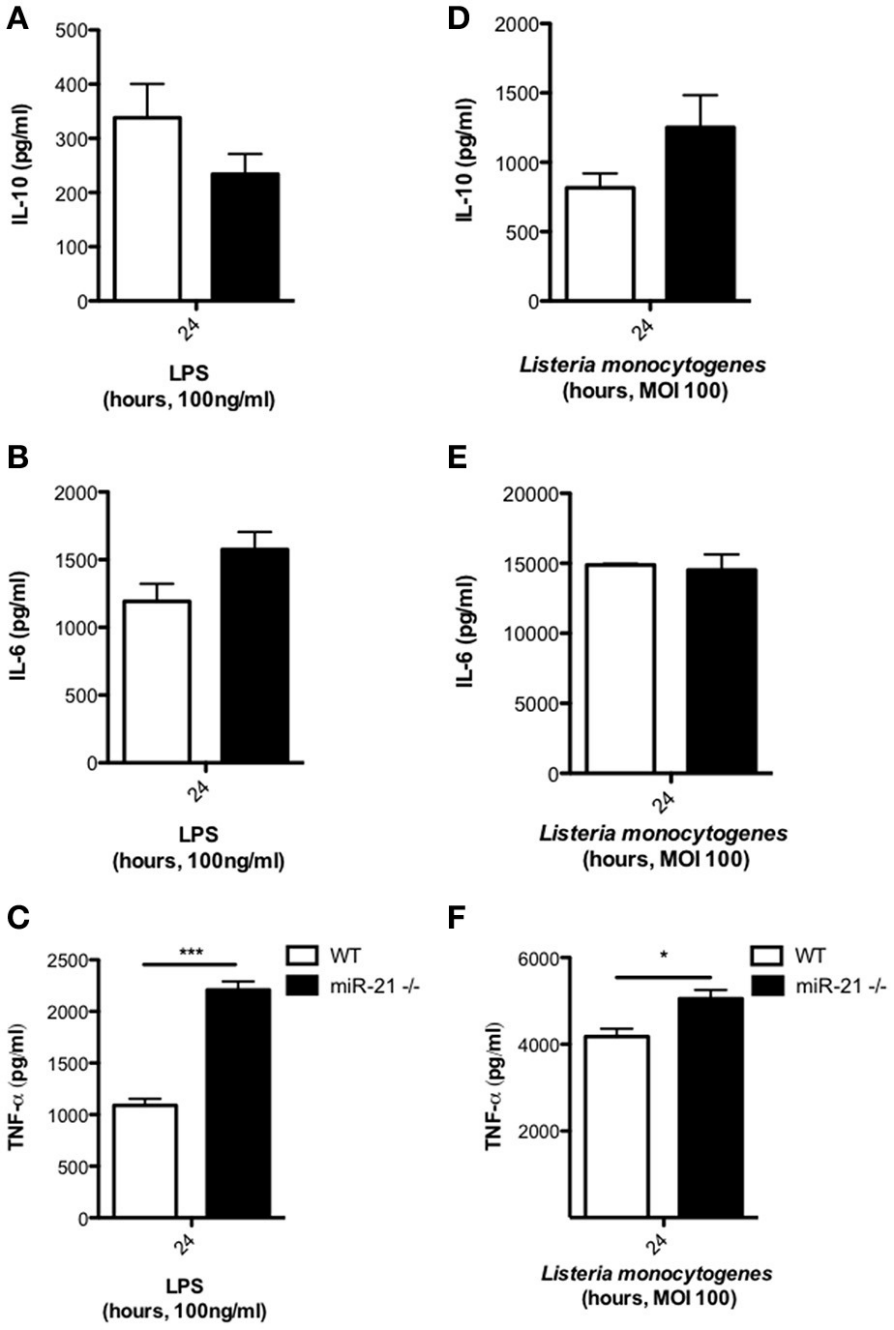
**MiR-21-deficiency alters cytokine response to LPS and *Listeria***. BMDMs were incubated with LPS at 100 ng/ml for 24 h and levels of **(A)** IL-10, **(B)** IL-6, and **(C)** TNF-α in the supernatants measured by ELISA. BMDMs were infected with *L. monocytogenes* at an MOI 100 for 15 min and subsequently cultured with media containing gentamicin (100 μg/ml) and at 24 h post-infection the levels of **(D)** IL-10, **(E)** IL-6, and **(F)** TNF-α in the supernatants measured by ELISA. Data are expressed as means ± SD, *n* = 3, and are representative of at least three independent experiments. ^*^Designates a *p-*value < 0.05 and ^***^Designates a *p-*value < 0.001 by Students *t*-test.

To assess the effect of miR-21 on the immunological response of macrophages during infection, we treated WT and miR-21^−/−^ BMDMs with LPS or infected with *L. monocytogenes*, and after 24 h determined the level of cytokine secretion. Although not significant, we observed a decrease in IL-10 secretion by miR-21^−/−^ cells in response to stimulation with LPS (Figure 2A). This is in agreement with previous studies implicating miR-21 in IL-10 regulation (Sheedy et al., 2010). Furthermore, we observed a trend toward an increase in IL-6 secretion (Figure 2B) and significantly increased TNF-α secretion (Figure 2C) in cells lacking miR-21. We next determined the effect of miR-21 expression on cytokine responses during infection of macrophages with *L. monocytogenes*. We observed an increase in IL-10 secretion, counter to previous reports involving TLR ligands, a very high level of IL-6 secretion in both WT and miR-21^−/−^ cells and a significant increase in TNF-α secretion by miR-21^−/−^ macrophages, though the difference was less significant than that present post LPS stimulation (Figures 2D–F). This suggests that miR-21's capacity to affect multiple processes may present a more complex dynamic during infection. This idea is supported by recent work by Barnett et al. who reported varying survival and cytokine secretion profiles among different sepsis models in miR-21^−/−^ mice, where the more physiological cecal ligation puncture model showed no differences between WT and miR-21^−/−^ mice in contrast to an LPS-induced sepsis model (Barnett et al., 2016).

### Loss of MiR-21 expression does not impact production of bacteriocidal nitric oxide in response to infection

**Figure 3:**
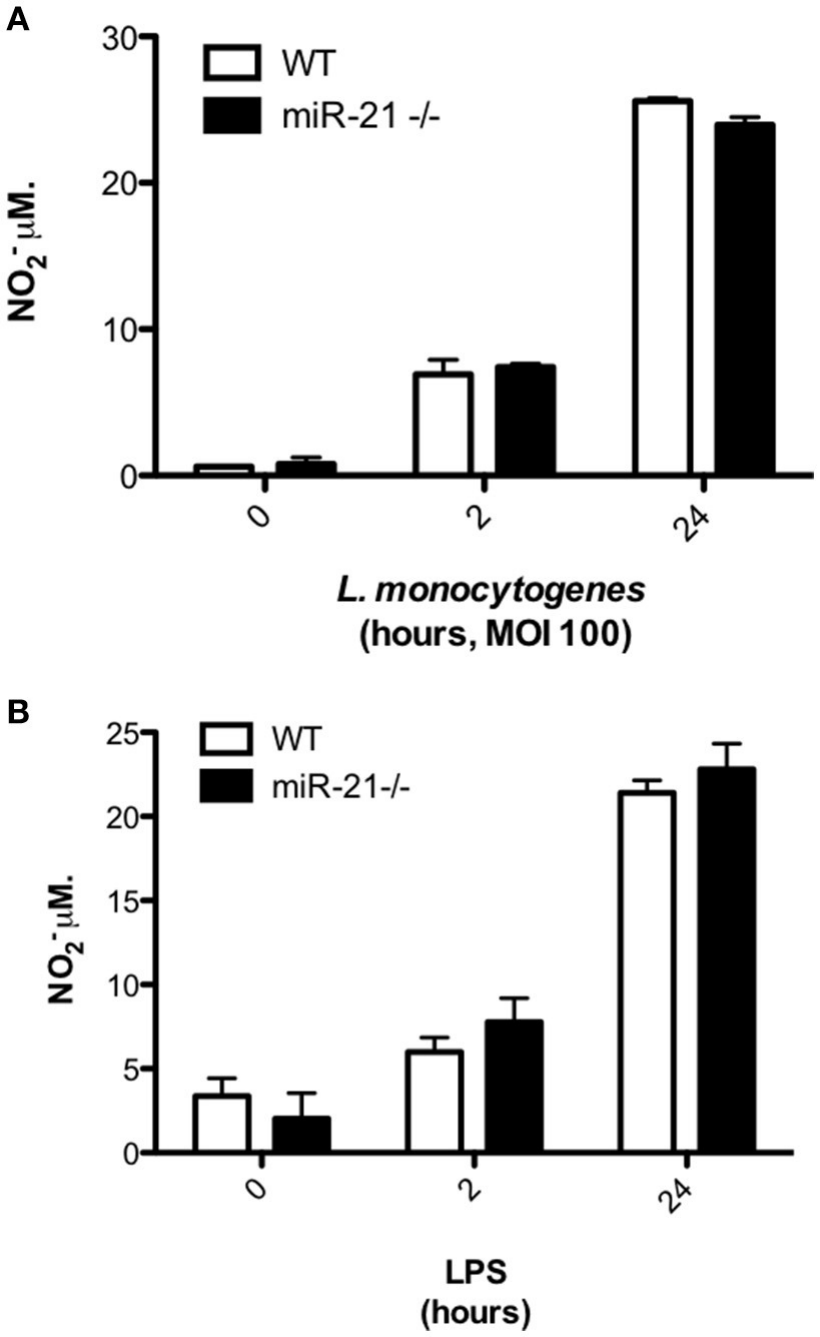
**MiR-21-deficiency does not impact on NO^−^ production in response to infection with *Listeria*. (A)** BMDMs were treated with LPS (100 ng/ml) before the supernatant was harvested and NO^−^ production was assayed by Griess reaction. **(B)** BMDMs were infected with *L. monocytogenes* at an MOI 100 for 15 min and subsequently cultured with media containing gentamicin (100 μg/ml). At 2 and 24 h post-infection the supernatant was harvested and NO^−^ production was assayed by Griess reaction.

We next explored the possibility that miR-21 induction may regulate the antibacterial activities of macrophages. As the oxygen-free radical NO is an important factor produced within macrophages to mediate intracellular killing of phagocytosed bacteria, we assayed the effect of loss of miR-21 expression on their production in response to infection with *L. monocytogenes*. To determine if reduced production of NO was responsible for the heightened bacterial load in miR-21^−/−^ BMDMs, we infected cells with *Listeria* and harvested the supernatants to measure nitrite using the Griess reaction. We saw no significant difference in NO production between WT and miR-21^−/−^ macrophages (Figure 3A). Similarly, we saw no significant difference in the production of NO by WT and miR-21^−/−^ macrophages in response to LPS (Figure 3B). These data led us to the conclusion that miR-21^−/−^ cells are not impaired in the capacity to kill invading bacteria and that the higher burden observed in these cells is due to miR-21 regulation of an alternative part of the phagocytosis process.

### MiR-21 limits uptake of FITC-dextran and FITC-*E. coli* by macrophages

**Figure 4:**
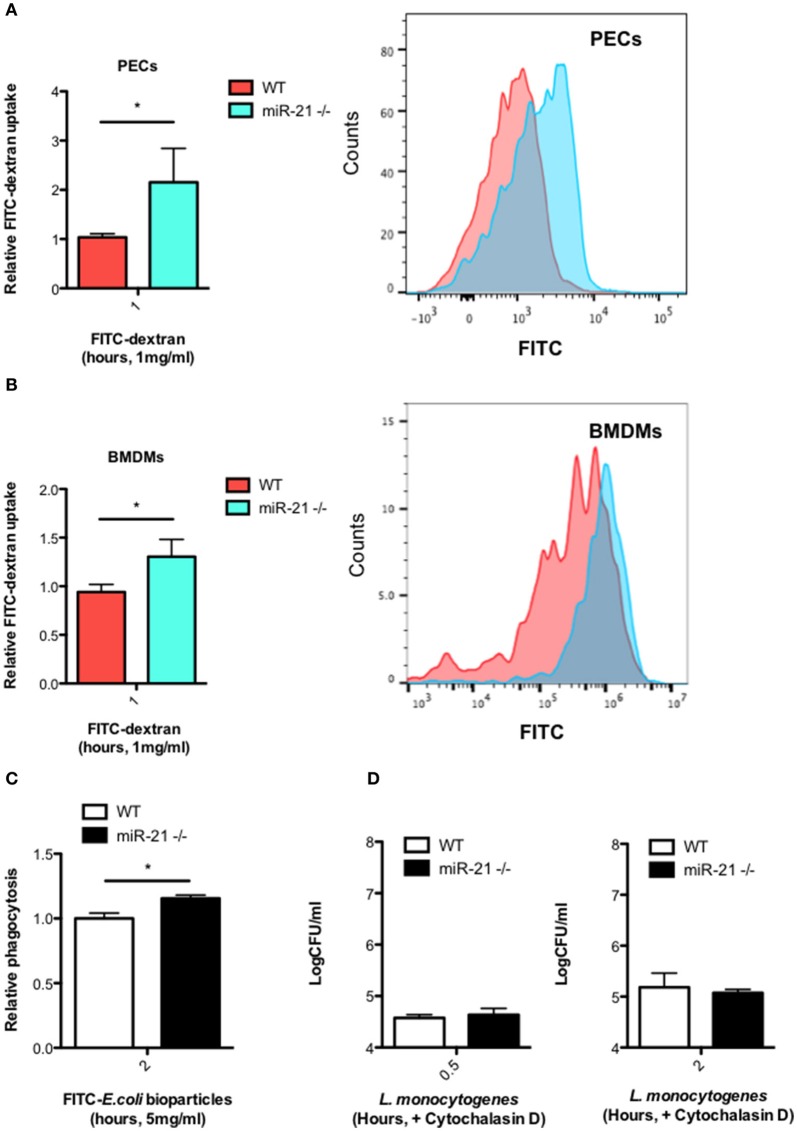
**MiR-21-deficient macrophages display increased uptake of FITC-dextran and phagocytosis of FITC-*E. coli* particles**. Peritoneal exudate cells (PECs) **(A)** and BMDMs **(B)** from WT and miR-21^−/−^ mice were incubated with media containing 1 mg/ml FITC-dextran for 1 h at 37°C. Uptake of FITC-dextran was determined by measuring the median fluorescence intensity by flow cytometry and data expressed relative to WT (Relative FITC-dextran uptake) with a corresponding histogram representative of median florescent intensity. **(C)** BMDMs from WT and miR-21^−/−^ were incubated with FITC-K-12 *E. coli* Bioparticles for 2 h before fluorescence was determined using a microplate reader. **(D)** WT and miR-21^−/−^ BMDMs were treated with cytochalasin-D for 30 min prior to *Listeria* infection. At 0.5 and 2 h post-infection, the number of intracellular bacteria was determined and expressed as Log CFU/ml. Data are expressed as means ± SD, *n* = 3 [except **(B)** where *n* = 2 for miR-21^−/−^ BMDMs], and are representative of at least two independent experiments. ^*^Designates a *p-*value < 0.05 by Students *t*-test.

Given that we observed an increased intracellular bacterial burden in miR-21^−/−^ cells as early as 30 min post-infection (Figure 1C), we hypothesized that miR-21 may regulate the initial engulfment of bacteria by macrophages. To determine this, we next examined the ability of resident peritoneal macrophages (PECs) (Figure 4A) and BMDMs (Figure 4B) to take up FITC-dextran. Cells were incubated with FITC-dextran for 1 h and its uptake assayed by flow cytometry. Interestingly, there was a significantly higher uptake of FITC-dextran in both PECs and BMDMs (Figures 4A,B) in miR-21^−/−^ cells compared to WT macrophages. As dextran is not necessarily taken up by phagocytosis, we also used a commercial phagocytosis kit to assay FITC-*E. coli* particle uptake by macrophages and saw that there was a significant increase in uptake by miR-21^−/−^ cells relative to WT (Figure 4C). Pre-treatment of cells with the actin polymerization inhibitor cytochalasin-D ablated the previously demonstrated increase in bacterial burden demonstrated post-*L. monocytogenes* infection at both 30 min and 2 h (Figure 4D). These results indicated that miR-21 may regulate the phagocytic process by mediation of actin polymerization.

### MiR-21 represses the pro-phagocytic proteins MARCKS and RhoB

**Figure 5:**
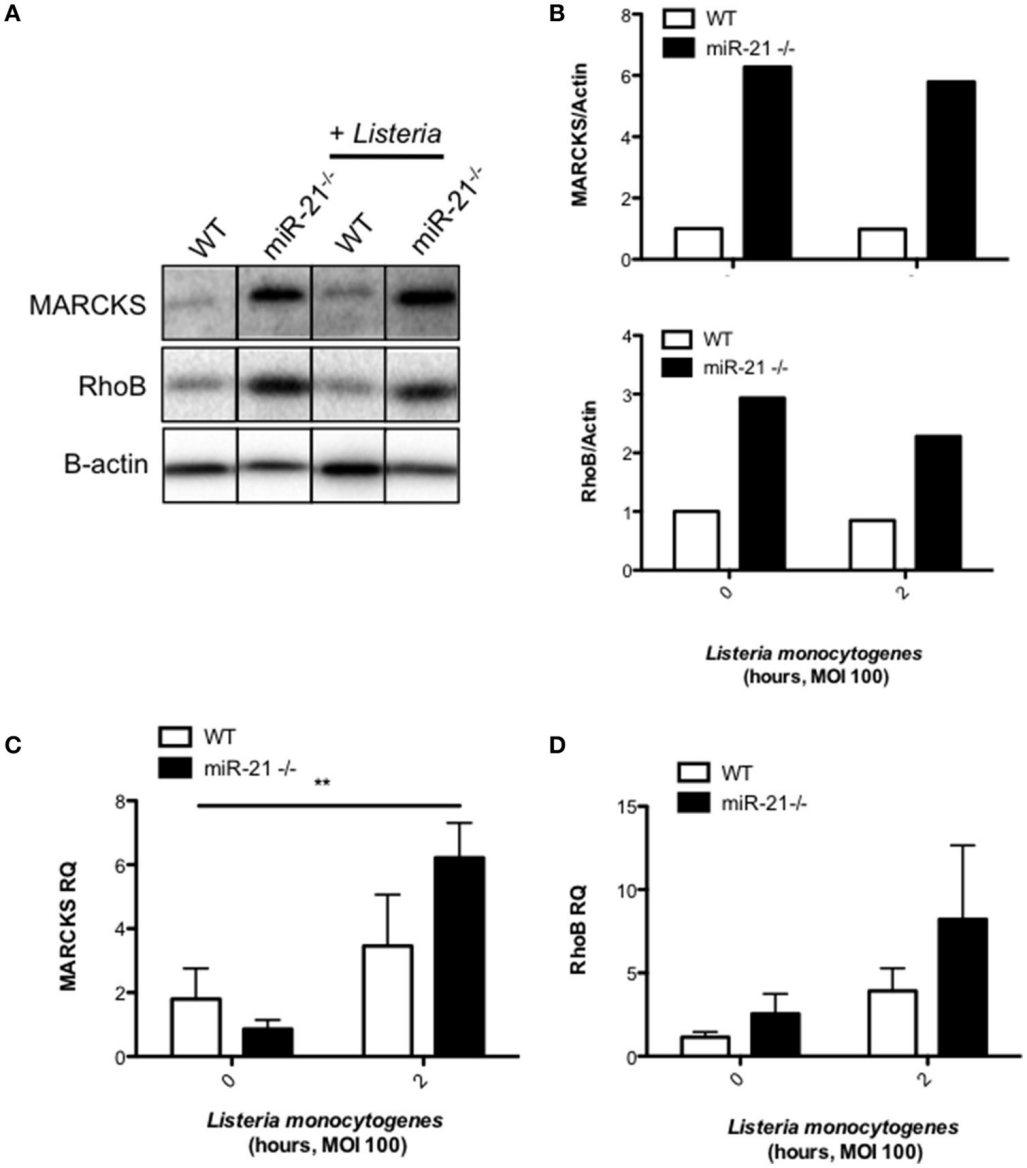
**MiR-21 represses the pro-phagocytic proteins MARCKS and RhoB. (A)** BMDMs were infected with *L. monocytogenes* at an MOI 100 for 15 min and subsequently cultured with media containing gentamicin (100 μg/ml) for a further 105 min. **(A)** At 2 h post-infection, protein lysates were prepared for immunoblot analysis of basal and post-infection MARCKS and RhoB protein amounts. Protein levels were quantified by densitometry and expressed relative to the endogenous control **(B)**. RNA was isolated and assayed by qPCR for **(C)** MARCKS and **(D)** RhoB expression using Rps13 as an endogenous control. Data are expressed as means ± SD, *n* ≥ 5, and are representative of at least two independent experiments. ^**^Designates a *p-*value < 0.01 by Students *t*-test.

We next analyzed various databases of predicted and validated miR-21 target genes for factors which may influence initial uptake during phagocytosis. We identified myristoylated alanine-rich protein kinase C substrate (MARCKS), which has been shown in other studies to regulate actin polymerization and formation of the phagocytic cup. We observed a higher basal protein level of MARCKS in miR-21^−/−^ BMDMs compared to WT macrophages (Figures 5A,B). Infection had no effect of the level of MARCKS protein in both WT and miR-21^−/−^ macrophages (Figures 5A,B). There was no difference in the level of MARCKS mRNA basally between WT and miR-21^−/−^ macrophages, however, infection increased the mRNA level of MARCKS in miR-21^−/−^ macrophages, and this increase was significantly higher than in WT macrophages following infection (Figure 5C). We next analyzed another target of miR-21, *Ras* homolog gene family member B (RhoB), which has previously been reported to influence cytoskeletal changes during phagocytosis (Zhang et al., 2005; Quinn et al., 2009). We observed that the protein level of RhoB is higher basally in miR-21^−/−^ BMDMs compared to WT macrophages (Figures 5A,B). Infection had no effect on the protein level of RhoB (Figures 5A,B). Although not significant, there was a trend toward increased RhoB at the mRNA level basally in miR-21^−/−^ macrophages and also following infection with *L. monocytogenes* (Figure 5D). Taken together these data suggest that miR-21 targets MARCKS and RhoB, demonstrating that miR-21 interacts with known actin mediators. Although further work is required to show this, it may be that this represents a host strategy to limit the intracellular niche of *L. monocytogenes*.

### MiR-21 controls intraperitoneal *Listeria* infection *in vivo*

**Figure 6:**
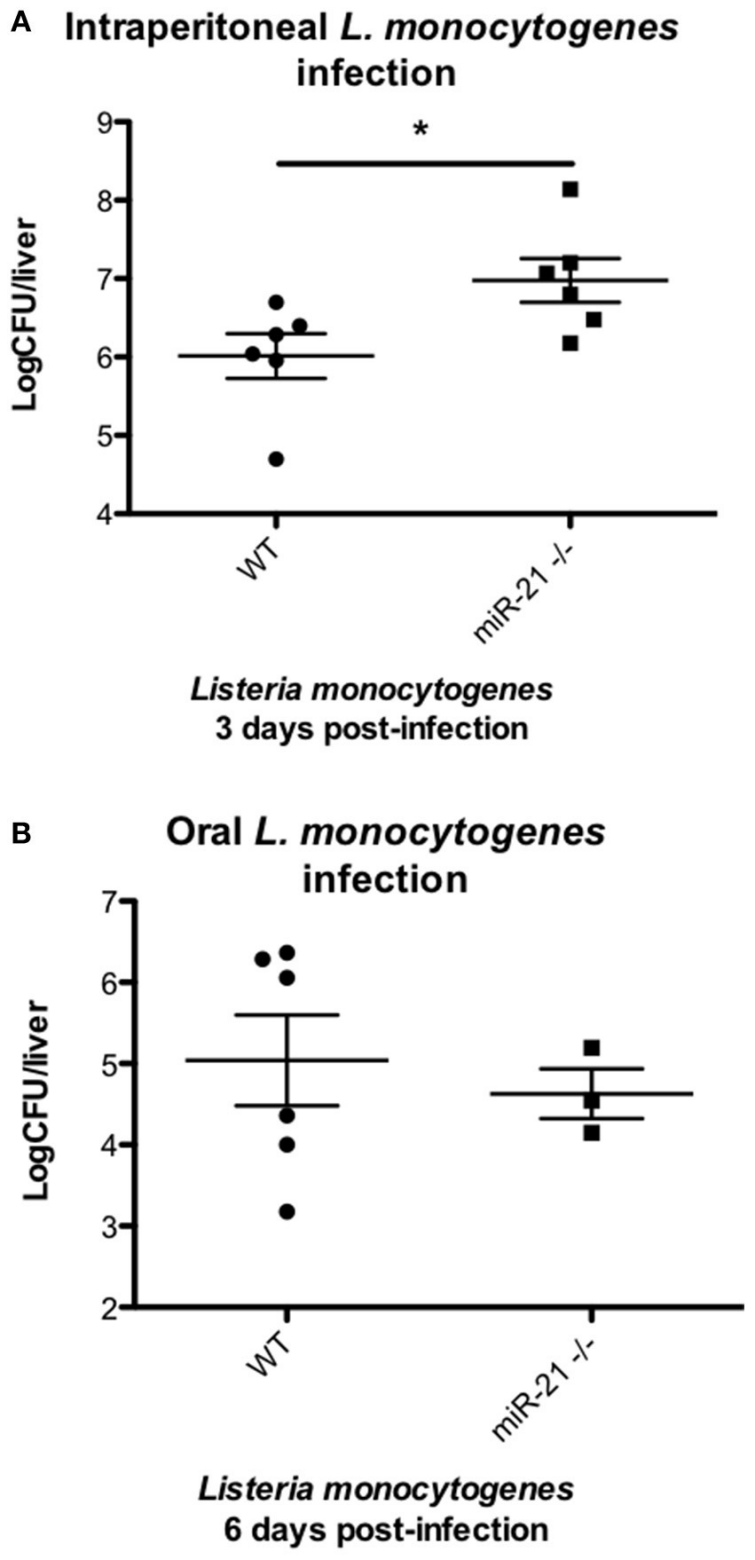
**miR-21-deficient mice are more susceptible to *Listeria* infection. (A)** Mice were intra-peritoneally infected with *Listeria monocytogenes* at 1 × 10^6^ CFU and 3 days post-infection dissemination of bacterial to livers determined and expressed as LogCFU/liver. Data are expressed as means ± SEM, *n* = 6. **(B)** Mice were fasted overnight before being orally infected with *Listeria monocytogenes* at 5 × 10^7^ CFU and 6 days post-infection dissemination of bacterial to livers determined and expressed as LogCFU/liver. Data are expressed as means ± SEM, *n* ≥ 3. ^*^Designates a *p-*value < 0.05 by Students *t*-test.

In order to test the hypothesis that induction of miR-21 during infection with *Listeria* is a host strategy to limit the intracellular niche of this bacterium, thereby reducing its pathogenesis and the ability of *L. monocytogenes* to establish an infection, we compared the outcome of intra-peritoneal infection of WT and miR-21^−/−^ mice. We observed reduced levels of dissemination by *Listeria* to internal organs, as shown by a significant reduction in bacterial burden in the livers (Figure 6A) of WT mice, compared to miR-21^−/−^ mice. This difference was not apparent in mice infected by oral gavage, which might indicate that the dissemination is a macrophage led phenotype (Figure 6B).

## Discussion

Macrophages form a crucial part of our body's defense, with the ability of macrophages to engulf and digest invading pathogens, termed phagocytosis, being fundamental to the control of infection (Rougerie et al., 2013). Bacterial pathogens are sensed through the expression of PRRs on phagocytes which recognize pathogen-associated molecular patterns (PAMPS) such as bacterial lipopolysaccharide, including TLRs expressed on the phagocyte cell surface and membranes of vesicular compartments (Janeway and Medzhitov, 2002; O'Neill et al., 2013) This results in the initiation of appropriate intracellular signaling pathways that trigger active phagocytosis and also production of cytokines to alert nearby immune cells (Kaufmann and Dorhoi, 2016). Control of bacterial pathogens is an important function of phagocytes, however certain intracellular bacteria have evolved strategies which allow them to survive within macrophages and exploit this intracellular niche. This enables propagation of infection without immune detection and activation of antimicrobial responses.

In this work, we have described a novel regulatory role for miR-21, whereby it limits this intracellular niche by inhibiting initial uptake of bacteria within macrophages, thereby reducing the severity and outcome of infection. We demonstrate here, that in the absence of miR-21, macrophages exhibit an increased bacterial burden following infection with the intracellular pathogen, *L. monocytogenes*. MiR-21 was induced following infection of BMDMs with *L. monocytogenes*. MiR-21 has previously been shown to be induced in response to bacterial LPS in macrophages while it is induced in monocytes infected with *Mycobacterium leprae* (Sheedy et al., 2010; Liu et al., 2012). We were surprised to find that miR-21-deficiency in BMDMs led to a higher bacterial burden post-infection which translated to increased bacterial dissemination and infection in mice following i.p. infection. These results are in contrast to previous studies showing an anti-inflammatory role for miR-21 and suggesting that macrophages with high miR-21 expression are more M2 like (Sheedy, 2015). This would imply that miR-21-deficiency should correspond to a more pro-inflammatory and bactericidal phenotype. Indeed our own cytokine data supports this model, providing evidence for the miR-21 being a negative regulator of pro-inflammatory responses by inducing anti-inflammatory IL-10, as well as supporting the pre-existing studies for miR-21's negative regulation of TNF-α (Sheedy et al., 2010; Barnett et al., 2016). However, while these observations were consistent with the purified ligand LPS they were not fully consistent in an infection setting when *Listeria* were used to stimulate the macrophages, though there was still a significant increase in TNF-α secretion in the miR-21^−/−^ macrophages. This failure of translation of ligand effect to physiological settings has been seen in other studies and points to a complexity in the role of the multi-target miR-21 in infectious settings (Barnett et al., 2016). In addition, the high bacterial burden present in our system may lead to an overwhelming cytokine signal, as demonstrated by the very high levels of the key *L. monocytogenes* cytokine IL-6 observed in response to infection (Dalrymple et al., 1995), which may hide potential differences.

Fusion of the phagosome with lysosomes, to form the phagolysosome is accompanied by acquisition of antibacterial mechanisms including antimicrobial peptides, proteases, an acidified environment, and the production of reactive oxygen and nitrogen species (ROS, RNS respectively), with NO (Rougerie et al., 2013). The production of NO during the oxidative burst which accompanies engulfment is a key antimicrobial mechanism of phagocytic cells, and has been shown to be important for the clearance of *L. monocytogenes* (Shiloh et al., 1999). To determine whether miR-21 may be influencing production of these antimicrobial molecules we determined production of NO in WT and miR-21^−/−^ BMDM in response to *Listeria* infection. NO which is produced via the enzymatic activity of inducible nitric oxide synthase 2, has been shown to be an important mediator of immune responses to *Listeria* (MacMicking et al., 1995; Endres et al., 1997). In our study, NO was produced at similar levels in WT and miR-21^−/−^ BMDM in response to both *L. monocytogenes* and LPS, indicating that the increased bacterial burden in miR-21-deficient macrophages is not due to a defect in production of RNS. It would be interesting to assess the role of ROS in this system, as miR-21 has been demonstrated to impact generation of these antibacterial mediators in cancer settings (Jiang et al., 2014; Guo et al., 2015).

As the increased bacterial burden in miR-21^−/−^ cells was observed as early as 30 min post-infection, we questioned whether miR-21 may be influencing the initial engulfment and uptake of bacteria by macrophages. By assessing uptake of FITC-dextran by miR-21^−/−^ and WT macrophages, we show that miR-21 influences the capacity for macrophages to take up fluid via pinocytosis or macropinocytosis. This was observed in both BMDMs and resident peritoneal macrophages. However, dextran uptake is not necessarily a phagocytic process (Pustylnikov et al., 2014) and so we sought to confirm using a phagocytosis-specific assay. We observed that miR-21^−/−^ BMDMs took up significantly higher levels of FITC labeled *E. coli* particles relative to WT BMDMs using a commercially available phagocytosis-specific kit. Phagocytosis and in particular initial uptake, is dependent primarily on the actin cytoskeleton, and a series of cytoskeletal rearrangements triggered following receptor activation. The most well characterized phagocytic receptors on macrophages include FcγRs and complement receptor 3 (CR3), which bind to immunolglobulin G (IgG)-opsonised particles and complement-coated particles respectively (Kerrigan and Brown, 2009). Key mediators of actin rearrangements are the RhoGTPases, which have been shown to be important for the initial formation of the phagocytic cup, which engulfs the invading pathogen (Rougerie et al., 2013). We identified several targets of miR-21 involved in phagocytosis and actin rearrangement including the RhoGTPase, RhoB, which has been reported to operate in coordination with Cdc42 (Allen and Aderem, 1995; Caron and Hall, 1998; Carballo et al., 1999; Corradin et al., 1999; Zhang et al., 2005; Quinn et al., 2009; Yang et al., 2013; Choi et al., 2015). We confirmed that RhoB is present in higher amounts in miR-21-deficient cells, and that it is induced to a higher degree in miR-21-deficient cells at the RNA level. Further work is required to fully elucidate the role of RhoB in this system.

Macrophages have been shown to express high levels of the myristoylated, alanine-rich, C kinase substrate (MARCKS), an actin cross-linking protein (Carballo et al., 1999). In particular, this increased MARCKS expression is found in areas of the cell where actin filaments associate with the plasma membrane, and its expression is associated with regulation of cell motility (Myat et al., 1997; Carballo et al., 1999). A study by Carballo et al. implicated MARCKS in the regulation of phagocytosis of zymosan, specifically, in the rate of initial uptake (Carballo et al., 1999). MARCKS has recently been shown to be a target of miR-21 in epithelial cells, where its expression influences mucin secretion (Vazquez-Boland et al., 2001; Li et al., 2009; Lampe et al., 2013). Given that macrophages also express MARCKS, we wondered whether miR-21 may target MARCKS thereby regulating initial uptake by phagocytes. Indeed, we observed increased protein levels of MARCKS in miR-21^−/−^ BMDM compared to WT cells. Furthermore, we show that mRNA levels of MARCKS are induced in miR-21^−/−^ BMDM following infection with *L*. *monocytogenes*. However, as with RhoB, further work is required to fully elucidate what role MARCKS might play in this system, particularly as it has been demonstrated that MARCKS and MacMARCKS deletion does not significantly impact phagocytosis *in vivo* or *in vitro* (Underhill et al., 1998).

Intracellular bacteria frequently allow their engulfment by macrophages so that they can shelter from components of the host immune system. Following internalization, intracellular pathogens utilize sophisticated strategies to avoid destruction by these cells, enabling them to overcome host cell defenses and replicate successfully. They block intracellular killing by inhibiting phagosome maturation, or express effector proteins which allow them to escape into the cytosol (Kaufmann and Dorhoi, 2016). Escape from the phagosome into the cytosol is an evasion strategy employed by *L. monocytogenes* to avoid immune detection. *L. monocytogenes* uses listeriolysin (Hly), a thiol-activated cholesterol-dependent cytolysin to form pores in the membrane of the phagosome allowing escape into the cytosol (Singh et al., 2008). Subsequently, *L. monocytogenes* induces actin tails through expression of an actin polymerizing protein, ActA that facilitate its propulsion through the cell cytosol toward the cell membrane, where it forms protrusions into neighboring cells allowing its internalization and facilitating cell-cell (Williams et al., 2012). As a result of these bacterial strategies, there is an even greater pressure for host measures to counteract these immune evasion mechanisms in order to clear the infection. The ability of miR-21 do reduce internalization of *L. monocytogenes* by macrophages significantly impacts the outcome of infection in mice, with miR-21^−/−^ mice displaying a significantly higher bacterial burden compared to WT mice. The increased dissemination of *Listeria* to livers of mice following intraperitoneal infection is in direct agreement with our previous observation that miR-21^−/−^ resident peritoneal macrophages display increased phagocytosis of particles. This observation appears to be macrophage-specific as oral gavage of WT and miR-21^−/−^ revealed no differences in bacterial dissemination. Peritoneal macrophages have been demonstrated to act as a *L. monocytogenes* reservoir, enhancing the infectious capability of this bacterium (Drevets, 1999). In this study, we present a novel role for miR-21 during the host response to intracellular bacterial infection, whereby miR-21 regulates the fundamental process of phagocytosis. We demonstrate that miR-21 limits the actin modulating proteins RhoB and MARCKS, and suggest that this may important in explaining the observed limitation of *L. monocytogenes* infection by WT macrophages compared to miR-21^−/−^ cells.

## Author contributions

SC conceived ideas and oversaw the research programme. DJ carried out the research. SC and DJ analyzed data and wrote the manuscript. MW, ZZ, and JK performed experiments. LO funded creation of the miR21-deficient mice and provided mentorship during the course of the research.

## Funding

This study was funded by a Starting Investigator Research Grant from Science Foundation Ireland (SFI) (grant number 11/SIRG/B2099) awarded to SC.

### Conflict of interest statement

The authors declare that the research was conducted in the absence of any commercial or financial relationships that could be construed as a potential conflict of interest.
