# Supplementary figures and images for: MicroRNA-21 Limits Uptake of Listeria monocytogenes by Macrophages to Reduce the Intracellular Niche and Control Infection
Source: Front Cell Infect Microbiol. 2017 May 23;7:201. doi: 10.3389/fcimb.2017.00201 (PMC5440467; doi:10.3389/fcimb.2017.00201)

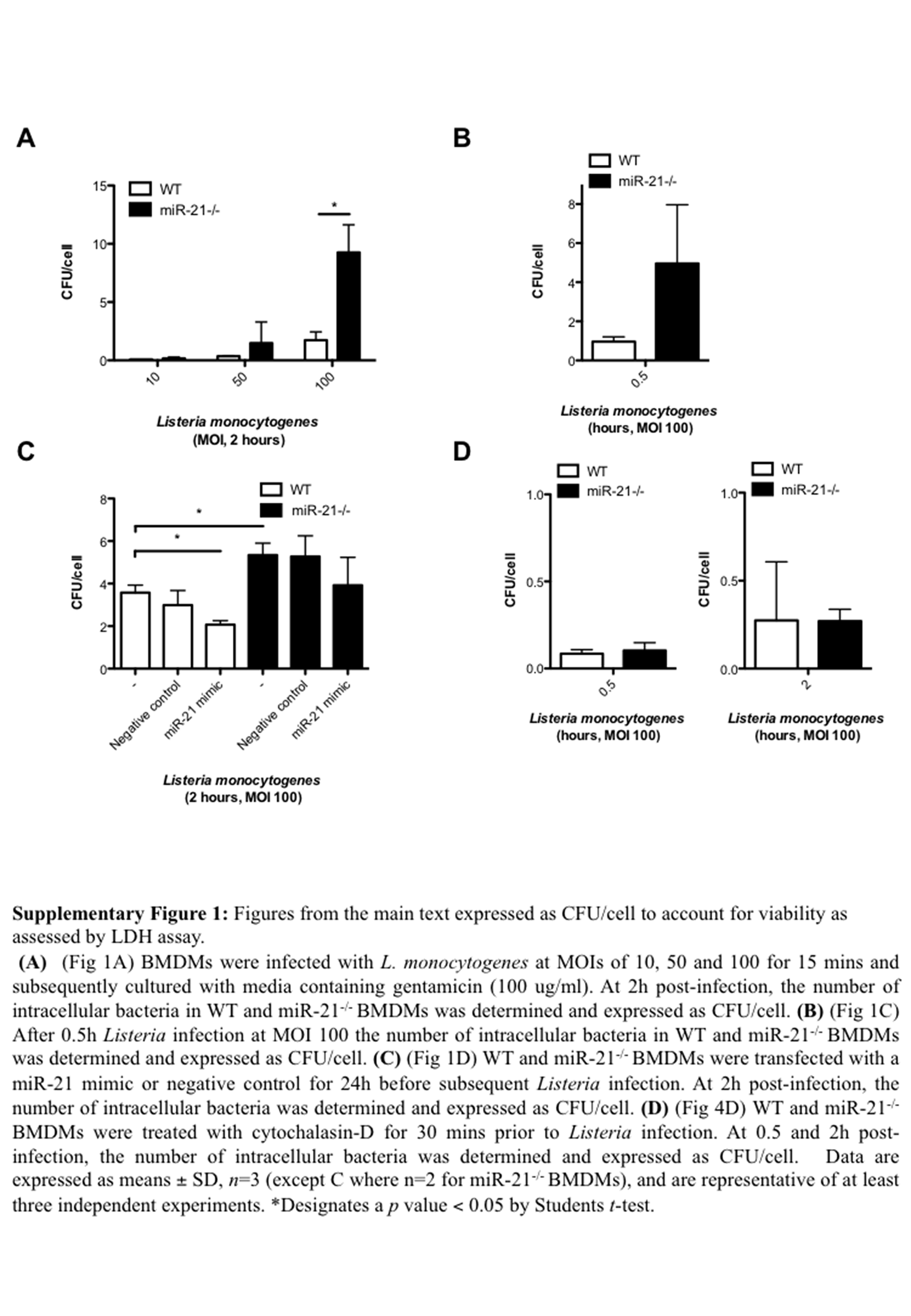

Supplement: Supplementary file 1 [file Image1.TIF]
